# Associations of state or trait dissociation with severity of psychopathology in young people with borderline personality disorder

**DOI:** 10.1186/s40479-023-00226-z

**Published:** 2023-06-12

**Authors:** Ashleigh P. Salmon, Katie Nicol, Michael Kaess, Martina Jovev, Jennifer K. Betts, Andrew M. Chanen

**Affiliations:** 1 Orygen, Melbourne, Australia; 2 Centre for Youth Mental Health, The University of Melbourne, Melbourne, Australia; 3 University Hospital of Child and Adolescent Psychiatry and Psychotherapy, University of Bern, Bern, Switzerland; 4 Department of Child and Adolescent Psychiatry, Center for Psychosocial Medicine, University Hospital Heidelberg, Heidelberg, Germany

**Keywords:** Personality disorder, Youth, Dissociation, Early intervention

## Abstract

**Background:**

State and trait dissociation are associated with borderline personality disorder (BPD) severity and severity of commonly co-occurring mental health symptoms. Although these distinct constructs do not consistently co-occur in experimental settings, they are frequently reported as the same construct, namely dissociation. This study aimed to investigate the co-occurrence of state and trait dissociation among young people with BPD and to examine whether state or trait dissociation were associated with symptom severity in this population.

**Methods:**

State dissociation was induced using a stressful behavioural task in a clinical sample of 51 young people (aged 15–25 years) with three or more BPD features. Diagnoses, state and trait dissociation, BPD severity and severity of posttraumatic stress disorder (PTSD), depressive, and stress symptoms were assessed by self-report or research interview.

**Results:**

A chi-square test of independence showed a strong association between state and trait dissociation. Bonferroni corrected *t*-tests showed that state dissociation was significantly associated with PTSD symptom severity and likely associated with BPD severity and severity of depressive and stress symptoms. Trait dissociation was not associated with symptom severity or severity of BPD features.

**Conclusions:**

These findings highlight the need to distinguish between state and trait dissociation in personality disorder research. They suggest that state dissociation might be an indicator of higher severity of psychopathology in young people with BPD.

## Introduction

Transient, stress-related dissociation is a diagnostic feature of borderline personality disorder (BPD) that was introduced in the Diagnostic and Statistical Manual of Mental Disorders (DSM) in its fourth edition (DSM-IV) [1] and has been retained in subsequent editions of the DSM. Dissociation is defined in the DSM as experiences of internal discontinuity or disruption in the normal integration of identity, consciousness, memory, perception, emotion, representation of the body, motor control, and behaviour.

Two meta-analyses of dissociation in people with psychiatric disorders have reported that only individuals diagnosed with posttraumatic stress disorder (PTSD) or dissociative disorders report higher rates of dissociation than among people diagnosed with BPD [2, 3]. Further, BPD usually co-occurs with other mental health diagnoses (‘comorbidity’) [4, 5] and dissociation among adults within BPD is associated with greater BPD severity [6, 7], higher symptom severity of co-occurring psychiatric disorders [8], and poorer responses to treatment [9].

A distinction is often made between state and trait dissociation. State dissociation is usually described as a transient symptom, lasting minutes, hours, or days [10]. Trait dissociation refers to relatively stable individual differences in dissociative experiences [1, 10]. Compared with other psychiatric disorders, trait dissociation is highly prevalent among people with a diagnosis of BPD. When present as a diagnostic feature of BPD, trait dissociation is usually exacerbated by stress [11].

Instances of state dissociation are also more common among individuals with BPD, compared with individuals with other psychiatric disorders or no psychiatric history [12, 13]. Like trait dissociation, state dissociation among adults with BPD is associated with higher levels of severity of both BPD and co- occurring disorders [7].

Trait dissociation is commonly measured using self-report and/or interviewer assessment [2, 3], while state dissociation is usually measured by self-report in response to dissociation-inducing stimuli or a stress-inducing behavioural task [14], or through real time monitoring of stress and dissociative symptoms [13]. While these constructs and their measurement are distinctly defined, state and trait dissociation are often conflated in the reporting of research findings under the term ‘dissociation’, which limits interpretation of past findings. Participants who report trait dissociation might not experience state dissociation in response to a specific behavioural stimulus, while those who report state dissociation in an experimental setting might not experience stress-related dissociative symptoms with sufficient frequency to meet the criteria for trait dissociation.

While state and trait dissociation have been measured in the same sample, few studies have compared separately the associations of these constructs with other variables within the same sample [15, 16].

Studies that do report state and trait dissociation separately suggest that each might be differentially associated with functioning and outcomes in BPD [17, 18]. However, this has not been specifically investigated with respect to BPD severity or severity of posttraumatic, depressive, or stress symptoms. Moreover, these studies have been conducted in samples of adults diagnosed with BPD, in whom state or trait dissociation might be influenced by ‘duration of illness’ factors, such as cumulative adverse experiences, treatment, and iatrogenic harm [19].

This study aimed to investigate separately the relationships between state or trait dissociation and symptom severity among young people with BPD features, early in the course of BPD. We hypothesised that: (1) not all individuals who experience trait dissociation will experience state dissociation, and that not all individuals who experience state dissociation will experience trait dissociation; (2) participants who endorse state dissociation will report greater BPD severity and higher severity of depressive, stress, and posttraumatic stress symptoms, compared with participants who do not endorse state dissociation; (3) participants who endorse trait dissociation will report greater BPD severity, and greater depressive, stress, and posttraumatic stress symptoms, than participants who did not endorse trait dissociation.

## Methods

### Participants and procedures

This study analysed existing data from a sample of 55 young people (aged 15–25 years) participating in a broader study examining the role of the hypothalamic pituitary adrenal axis in stress responses among young people with features of BPD. Young people with full-syndrome or subthreshold BPD were recruited from Orygen, a government-funded outpatient youth mental health service in north-western and western metropolitan Melbourne, Australia. See Table 1 for gender and co-occurring diagnoses in this sample.

Four participants were excluded during analysis due to incomplete data, giving a final sample of *N* = 51 (age *M*(*SD*) = 19.94 (2.90)). The study was granted ethical approval by the Melbourne Health Human Research Ethics Committee (HREC 2011.172).

Participants were included if they had three or more BPD features, fluency in English, and were able to provide written informed consent. Participants were excluded if they met criteria for a lifetime psychotic, bipolar I or bipolar II diagnosis or if they reported a medication or illness that could impact salivary cortisol secretion, a body mass index < 18 kg/m2, or a current or recent pregnancy (within the past month). All participants provided written informed consent before enrolling in the study.

**Table 1:** Gender and prevalence of co-occurring diagnoses within the sample

	*n* (%)
**Gender**	
Female	38 (74.5%)
Male	13 (25.5%)
**Mental state disorder diagnoses**	
Current mood disorder	26 (51.0%)
Past mood disorder	22 (43.1%)
Current anxiety disorders	26 (51.0%)
Past anxiety disorders	5 (9.8%)
Current eating disorders	5 (9.8%)
Past eating disorders	3 (5.9%)
**Personality disorder diagnoses**	
Full threshold BPD (≥ 5 traits)	27 (52.9%)
Subthreshold BPD (3–4 traits)	24 (47.1%)

Demographic data were collected, and diagnoses and BPD severity were assessed by trained graduate research assistants, overseen by the principal investigator and project manager.

**Fig. 1:**
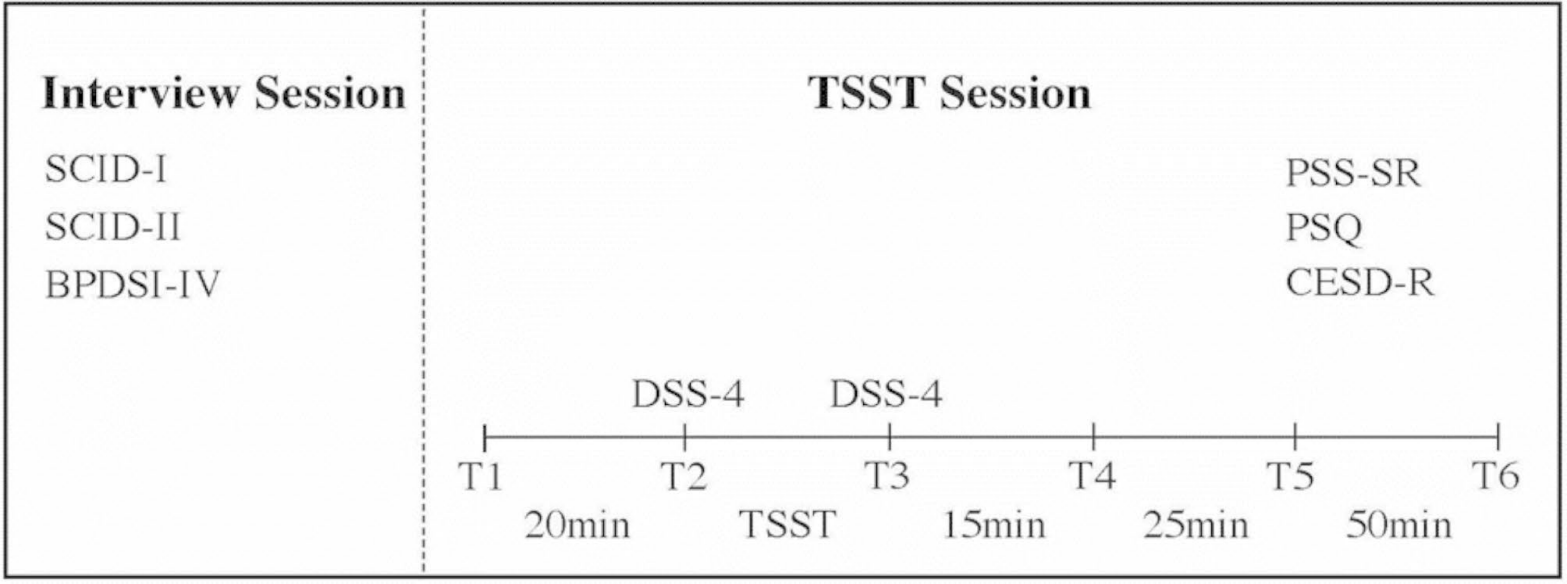
Timing of the administration of all study measures

Eligible participants were invited to return on another day to complete the Trier Social Stress Task (TSST) [20] and self-report measures of dissociative experiences and symptom severity for perceived stress, posttraumatic stress, and depressive symptoms. The TSST is a standardised psychosocial stress protocol that requires participants to complete a five-minute mock job interview and a five-minute mental arithmetic task in front of an audience of two panel members. Participants were introduced to the mock job interview task ten minutes before commencing the TSST and given this time to prepare. The TSST reliably induces a physiological stress response in anticipation of the stress task (after receiving instructions), and during completion of the stress task. During the TSST session, participants completed self-report assessments at six timepoints; upon arrival (T1), after receiving instructions for the ask (T2), and at four timed intervals after completing the task (T3-T6). See Fig. 1. for the timing of data collection for each measure.

### Measures

#### Diagnoses

Mental state disorders were assessed using the Structured Clinical Interview for the DSM-IV-TR Axis I Disorders Patient Edition (SCID-I/P) [21]. BPD criteria were assessed using the Structured Clinical Interview for the DSM-IV (SCID-II) [22]. The SCID-I/P and SCID-II have moderate to excellent inter-rater reliability [23].

#### Dissociation

State dissociation was measured with the Dissociation-Tension Scale-4 (DSS-4) [24] at T2, after receiving instructions for the task (T2) and at T3, after completing the task, reflecting the time points during which stress was induced.The DSS-4 is a four item, ten-point self-report scale with good to excellent internal consistency, reliability, and differential validity [24]. It has been validated as sensitive to changes in dissociative symptomatology over short periods of time [24]. A binary measure of state dissociation was derived for the primary analyses. A mean score (DSS-4_T2T3_) was calculated from DSS-4 scores reported at T2 and T3. State dissociation was classified as present if DSS-4_T2T3_ ≥ 1 and absent if DSS-4_T2T3_ < 1.

Participants were defined as experiencing trait dissociation if they were rated as experiencing threshold or subthreshold transient stress-related dissociation as defined in criterion 9 of the SCID II. Prior to analysis, authors identified 29 participants with an interviewer rating of threshold or subthreshold (i.e. a score of 2 or 3) on criterion 9. This rating was assessed by a second rater to determine whether participants experienced dissociation, paranoia, or both. Consensus was sought with a third rater for ambiguous cases. Two participants with threshold or subthreshold ratings for criterion 9 were not classified as experiencing dissociation as they reported only threshold or subthreshold paranoia for this item. Two participants were excluded as raters could not determine whether dissociation was present.

#### BPD severity and symptom severity

BPD severity was assessed with the Borderline Personality Disorder Severity Index-IV (BPDSI-IV) [25] a 70- item semi-structured interview which assesses the frequency and severity of BPD over the preceding three months. The five dissociation items were removed from the total score to avoid multicollinearity.

PTSD, stress, and depressive symptom severity were measured using three self-report measures: (1) the PTSD Symptom Scale – Self-Report version (PSS-SR) [26], (2) Perceived Stress Questionnaire (PSQ) [27], and (3) the Center for Epidemiologic Studies Depression Scale—Revised (CESD-R) [28].

Each measure has been well-validated. Psychometric evaluations have demonstrated: good concurrent validity, high test-retest reliability, and satisfactory internal consistency in the PSS-SR [26]; good test-retest reliability, internal consistency, and predictive validity in the PSQ [27]; high internal consistency and good convergent and divergent validity in the CESD-R [29]; and excellent inter-rater reliability, high to very high internal consistency, and very good discriminant, concurrent, and construct validity in the BPDSI-IV [25].

### Data analysis

All continuous variables (severity scores and DSS-4_T2T3_) were assessed for normality using descriptive statistics, the Shapiro-Wilk test and visual inspection of the distribution. Severity scores were normally distributed. However, a substantial violation of normality was observed in the DSS-4_T2T3_ scores. Substantial positive skew arose from a large cluster of tied scores at the lowest end of the DSS-4 scale; 27 participants (52.9%) reported no perceived state dissociation (DSS-4_T2T3_ = 0) at either timepoint, and 10 participants reported a DSS-4_T2T3_ score between 0 and 1. Non-normality could not be corrected through transformation. Non-monotonic associations were observed between state dissociation and each measure of severity, using ranked scores. Hence, authors chose to categorise the continuous DSS-4_T2T3_ variable, using a binary classification (absent or present). The sample size was insufficient to derive a categorical variable indicating the degree of state dissociation observed. This approach is considered appropriate for highly skewed data with distributions of this shape [30] and similar methods have been used previously in studies investigating state dissociation in BPD [31]. In the absence of a validated clinical threshold, authors’ selected a cut-off score with consideration to group size and the authors’ judgement of the minimum score required to suggest meaningful state dissociation.

A chi-square test of independence was conducted to examine the relationship between trait and state dissociation within the sample. Expected cell values were calculated to ensure the sample size was adequate [32]. Subsequently, eight independent sample *t*-tests were used to compare BPD severity, and PTSD, stress, and depressive symptom severity between participants who did and did not experience dissociation for both state and trait measures of dissociation. A Levene’s test confirmed homogeneity of variances for each analysis. A Bonferroni corrected threshold for significance was calculated to account for multiple comparisons [33]. For each independent-samples *t*-test, Cohen’s *d* was calculated to measure effect size.

## Results

A chi-square test of independence found a significant association between trait and state dissociation χ^2^(1, *N* = 51) = 5.4, *p* = 0.02. However, trait and state dissociation did not perfectly co-occur in each participant. Among the sample, 25.5% of participants met criteria for trait dissociation but did not report state dissociation during the task, while 7.8% of participants reported state dissociation during the task but did not meet the criteria for trait dissociation (see Table 2).

**Table 2:** Observed prevalence and co-occurrence of state dissociation (DSS-4) and trait dissociation (SCID-II)

		Trait dissociation (SCID-II)		
		Absent	Present	Total
**State dissociation (DSS-4)**				
	Absent	24 (47.1%)	13 (25.5%)	37 (72.5%)
	Present	4 (7.8%)	10 (19.6%)	14 (27.5%)
Total		28 (54.9%)	23 (45.1%)	51 (100%)

A series of independent-samples *t*-tests found that participants who endorsed state dissociation during the behavioural task reported significantly higher BPD severity and higher severity of PTSD, depressive and stress symptoms (see Table 3). After correcting for multiple comparisons, only stress severity remained significantly different between groups.

**Table 3:** Observed mean differences in BPD severity and related symptom severity

		Dissociation							
		Absent	Present					95% CI^a^	
		*M* (*SD*)	*M* (*SD*)	*t*	*df*	*p*	*d*	Lower	Upper
**State dissociation**									
	*CESD-R*	36.22 (11.21)	43.64 (9.35)	-2.20	49	0.032*	-0.69	-14.21	-0.65
	*PSQ*	83.99 (12.10)	92.37 (12.62)	-2.18	49	0.034*	-0.68	-16.10	-0.67
	*PSS-SR*	18.71 (9.82)	28.50 (11.29)	-3.05	49	0.004**	-0.96	-16.24	-3.34
	*BPDSI-IV*	22.36 (9.97)	29.65 (12.45)	-2.18	49	0.034*	-0.68	-14.03	-0.56
**Trait dissociation**									
	*CESD-R*	38.29 (2.05)	38.22 (2.45)	0.02	49	0.98	0.01	-6.30	6.44
	*PSQ*	86.56 (2.19)	85.97 (2.96)	0.16	49	0.87	0.05	-6.66	7.84
	*PSS-SR*	19.75 (2.08)	23.41 (2.29)	-1.18	49	0.24	-0.33	-9.88	2.56
	*BPDSI-IV*	22.37 (1.93)	26.78 (2.46)	-1.43	49	0.16	-0.40	-10.60	1.79

Note: * indicates significance at *p* < 0.05,

** indicates significance after Bonferroni correction (*p* < 0.00625),

^a^ 95% CI = 95% confidence interval for the population mean difference

For state dissociation, a large effect was observed for the estimated mean difference in PTSD symptom severity scores and moderate effect sizes were observed for estimated mean differences in BPD severity and depressive and stress symptom severity scores. No significant differences were found between participants that were and were not assessed as experiencing trait dissociation (see Table 3). For trait dissociation, effect sizes for the mean difference in severity scores on each domain, ranged from small to negligible.

## Discussion

Two key findings arise from this study of trait and state dissociation among young people with BPD features, who were presenting for treatment early in the course of the disorder. First, although there was a significant relationship between state and trait dissociation, these did not co-occur in every participant, suggesting that state and trait dissociation are related but not identical constructs. Second, state dissociation was significantly associated with PTSD. While associations were observed between state dissociation and BPD severity, depression, and anxiety, these were non-significant following correction for multiple comparisons. No associations were observed between trait dissociation and any other measure.

One third of participants reported experiencing either trait or state dissociation, but not both. This suggests that state and trait dissociation are distinct psychological constructs that should be investigated and reported separately in future studies examining the role of dissociation in BPD.

Participants who experienced trait dissociation did not differ in severity of BPD features or severity of PTSD, depressive, or stress symptoms, compared with participants who did not experience trait dissociation. Conversely, our findings offer evidence that participants who reported state dissociation in response to a stress-inducing behavioural task, also reported high levels of severity in all other measured domains. After correcting for multiple comparisons, the threshold for a statistically significant difference between these groups was only reached for PTSD symptom severity. However, the confidence intervals and large to moderate effect sizes suggest that state dissociation likely predicted greater symptom severity for each measure and that this effect might have been masked due to the relatively small sample size. Overall, these findings indicate that state and trait dissociation might be differentially associated with symptom severity among young people with BPD features, such that state dissociation is more strongly associated with BPD and co-occurring symptom severity than trait dissociation.

The finding that trait dissociation did not predict severity on any measure in this study, contradicts previous findings that trait dissociation is associated with higher reports of psychiatric symptom severity and comorbidity with other psychiatric disorders [6–8]. Previous studies have primarily analysed self- reported trait dissociation using the Dissociative Experience Scale [10].The current, novel finding might be explained by the presence of responder bias in self-report measures. Alternatively, the current findings might suggest that transient stress-related dissociation, as defined in DSM Criterion 9, does not capture the full range of dissociative experience in BPD. Perhaps more importantly, Criterion 9 fails to recognise dissociative experiences that are associated with significant levels of psychopathology among young people with BPD. This might reflect the early stage of BPD in the current sample and suggest that duration of illness factors might account for this association later in the course of BPD.

High rates of dissociation in PTSD [2] and high rates of co-occurring PTSD and BPD [34, 35] might account for the strength of evidence for this association in this study. Although the current study assessed diagnostic criteria for PTSD using the SCID I/P, the sample size did not allow for meaningful further analysis. To investigate the potential bias from co-occurring PTSD, future studies of trait and state dissociation in BPD should stratify the study population by presence/absence of PTSD diagnosis.

This study has several limitations. Authors analysed an existing dataset collected to address a different research question. As the sample size was fixed, no power analysis was conducted and the sample may have been insufficient for the reliable detection of small to medium effects. Similarly, criterion 9 of the SCID-II was the only available measure of trait dissociation for this sample. A secondary review of these data was necessary to discriminate trait dissociation from paranoia, thereby increasing the risk of measurement error. In similar studies, the SCID-II has been used infrequently, relative to other measures of trait dissociation [2]. While this reduces the external validity of these findings with existing literature, it also raises the issue of how trait dissociation might be assessed in future studies. While this reduces the external validity of these findings with existing literature, it also raises the issue of how trait dissociation might be assessed in future studies. Heightened subjective experiences are common in BPD and have been observed in other studies of young people with BPD regarding distress [36] and sleep [37], as well as among adults with BPD experiencing depression. Finally, state dissociation is difficult to reliably induce in experimental paradigms [38]. This study observed a low prevalence of state dissociation, leading to substantial skewness in the distribution of DSS-4 scores. To account for this, it was necessary to derive a binary measure of state dissociation using a cut-off value selected by the authors. This approach increased the risk of spurious findings in this study [39], and further replication is required to confirm these findings.

## Conclusions

This study offers preliminary evidence that state and trait dissociation are differentially associated with severity of psychopathology in young people with BPD. It also suggests that state and trait dissociation should be reported as distinct constructs in psychiatric research. Clinically, these findings suggest that reported instances of stress-related, state dissociation among young people with BPD might be a better indicator of severity of BPD and commonly co-occurring mental health symptoms, than trait dissociation, as defined in the DSM-IV and the fifth edition of the DSM (DSM-5) Section II.

## Data Availability

Data will be made available for reasonable use upon request to the corresponding author.

## Abbreviations

BPD: Borderline personality disorder
BPDSI-IV: Borderline Personality Disorder Severity Index-IV
CESD-R: Center for Epidemiologic Studies Depression Scale—Revised
DSM: Diagnostic and Statistical Manual of Mental Disorders
DSM-IV: Diagnostic and Statistical Manual of Mental Disorders, fourth edition
DSM-5: Diagnostic and Statistical Manual of Mental Disorders, fifth edition
DSS-4: Dissociation-Tension Scale-4
PSQ: Perceived Stress Questionnaire
PTSD: Posttraumatic stress disorder
PSS-SR: PTSD Symptom Scale – Self-Report version
SCID-II: Structured Clinical Interview for the DSM-IV
SCID-I/P: Structured Clinical Interview for the DSM-IV-TR Axis I Disorders Patient Edition
TSST: Trier Social Stress Task
